# Soccer above all? Analysis of academic and vocational education among female soccer players in the German women's Bundesliga and 2nd women's Bundesliga

**DOI:** 10.3389/fspor.2024.1294803

**Published:** 2024-02-12

**Authors:** Peter Ehnold, Andreas Gohritz, Lena Lotzen, Torsten Schlesinger

**Affiliations:** ^1^ Department of Sport & Management, IST-University of Applied Sciences Düsseldorf, Düsseldorf, Germany; ^2^ Institute of Human Movement Science and Health, Chemnitz University of Technology, Chemnitz, Germany

**Keywords:** women’s soccer, elite sport, women’s Bundesliga and 2nd women’s Bundesliga, vocational and academic education, influencing factors, logistic regression

## Abstract

**Introduction:**

Career-related (financial) reasons as well as advantages in terms of expanding social support systems, promoting a balanced lifestyle and personal development suggest that female soccer players should pursue academic or vocational education in parallel to elite sport. However, dual careers are fraught with challenges, mainly due to simultaneity in time and the associated conflicting goals. The aim of this article is to analyze the vocational or academic educational careers of professional female soccer players.

**Methods:**

To generate the data, an online survey was conducted among soccer players in the German Women's Bundesliga and 2nd Women's Bundesliga. A total of n = 200 questionnaires (German: *n* = 191; English: *n* = 9) were included in the analysis, which corresponds to approx. 29.6% of the population addressed.

**Results:**

90.6% of the players are pursuing or have already completed academic or vocational education. The majority (71.2%) of female soccer players choose to study. 81.8% of players report no impact or even a positive impact of soccer on their performance in academic or vocational education. Willingness to pursue and complete academic or vocational education is influenced by membership of the A-National Team, time spent playing soccer, form of school-leaving qualification, nationality and age.

**Discussion:**

This study increases the visibility of professional women's soccer as an object of analysis in sports science research, follows up on demands for a more athlete-centered approach and generates further insights for research and practice with regard to the success of dual careers in elite sport.

## 1 Introduction

Elite women's soccer is one of the most dynamically growing sports in the world (1). However, women's soccer has much lower income returns compared to men's soccer (2). Exceedingly few players will therefore succeed in securing their post-sport career financially in the long term through income earned during their soccer career (3). Thus, for most female soccer players, a career start or change will be necessary after the end of their top-level sporting career. It should be noted that the skills acquired in soccer are only conditionally transferable to the requirements in most occupational fields. This means that, at the end of a sports career, the (sports-related) human capital is devalued and can only be transferred to non-sports occupational fields to a limited extent (4). A successful transition from a sports career to a professional career after sport is therefore likely to depend to a large extent on whether female soccer players are able to adapt to the demands of the labor market in good time through appropriate further education. Adequate qualifications are indispensable for successfully making the transition to the regular labor market. By taking advantage of vocational or academic qualification offers, breaks in career and life trajectories can be prevented or at least reduced (5). In this respect, female soccer players should already think about their professional career after sport during their sports career in order to avoid the danger of falling into “professional nothingness” after the end of their soccer career (4).

However, vocational or academic certificates and experience can only be acquired if sufficient time resources are available. The choice of (further) vocational or academic education is made more difficult by the simultaneity of careers in sport and education and the associated conflicts of objectives regarding limited time resources (6). The challenges of a dual career that combines elite sport and vocational or academic education increase in comparison to dual careers of elite sport and school (7).

The aim of this article is to analyze vocational or academic education among professional women soccer players in the German Women's Bundesliga and 2nd Women's Bundesliga. The focus is on the following research questions: (*1*) *To what extent do elite female soccer players have started or already completed vocational or academic education during their sporting career? (2) To what extent does a sporting career influence vocational or academic education? (3) What factors influence the willingness for starting and completing vocational or academic education?.*

## 2 State of research

Studies on educational and professional careers in soccer mainly refer to the male sector. The focus is on the analysis of post-sport career paths in particular. Barth et al. (8) show in a systematic review that previous studies focus on health-related aspects and psychological problems after the end of a career (9–14). The aspect of occupational mobility (promotion and relegation) is also addressed in previous studies (15, 16). Furthermore, studies are available that address financial competencies (17, 18), the financial transition after the end of a career (19) and consumer and savings behavior (20, 21), as well as professional soccer as risks for the post-sporting career (22). In addition, statistics show that only around half of male soccer players in the third and fourth leagues in Germany pursue vocational education or already have a vocational or academic qualification (23).

For professional women's soccer, a Danish study shows that female soccer players have strong difficulties in balancing top-level sport with education, work and family commitments. As a consequence, these problems lead to an increased risk of early exit from soccer (24). A study of Canadian and Norwegian female soccer players shows that it is also difficult for female soccer players to find a career within soccer (for example, as a coach or in management) after their sporting career ends (25). Using the example of English female soccer players, it could be clarified that they prefer dual careers to a large extent, whereby the focus is placed on studying. The players cite the factor of “security” for their post-sport career as the main reason for pursuing a dual career (7).

Outside of soccer, there are many studies that deal with the topic of dual careers. As Vidal-Vilaplana et al. (26) present in a review, more than 100 articles on the topic of dual careers among elite athletes have been published in the period since 2017. A brief narrative overview of research on dual careers in elite sport and vocational or academic education outside soccer shows the following: existing studies address the compatibility of sporting demands and further education-related concerns of athletes (27–32). Aquilina (27), for example, shows in a cross-national comparative study that a university education and sporting success are quite compatible or can complement each other synergistically. Mutual benefits of pursuing a dual career are seen by the athletes, for example, in the fact that pressure and frustration in sport can be reduced through educational success and, vice versa, pressure and frustration in university education can be reduced through sporting success. Furthermore, athletes report that physical challenges can be better overcome through intellectual stimulation and that skills learned in one area can be transferred to the other. In addition, dual careers can lead to athletes showing a stronger sense of “balance” and therefore make them more motivated to continue their elite sporting career in the long term (27). On the other hand, the time-consuming aspect of pursuing an educational qualification alongside a sporting career should not be neglected. If these areas of life are not well coordinated, and if the athlete is not able to combine both areas in such a way that the burdens are well-balanced and acceptable for them, this can lead to increased stress, impaired mental health, burnout or cause them to drop out (33, 34).

Further studies examine the influence of mechanisms and factors on the process of dual careers of athletes. In addition to the handling of time compatibility (6, 35), factors that both enable and prevent top athletes from realizing dual careers are analyzed (32, 35–37). The flexibility of educational institutions and the stretching of vocational or academic education over time are also examined (38, 39).

Complementary studies address experiences in the context of dual careers and their prioritization (40–45). In terms of typologies of dual careers, a basic distinction is made between athletes who (a) focus equal attention on their sporting career and educational/vocational career, (b) prioritize their sporting career or (c) focus exclusively on sport (46). This distinction was extended by including career identity, athletic identity and self-efficacy in the analysis, whereby athletes who prioritize education or vocation could also be typologized (42). In addition, the holistic objective life situation of elite athletes is analyzed in further studies by using assessments of the socioeconomic system (athletic, educational, vocational, and financial) for typification (45).

Reflecting on the state of research, it becomes clear that although many studies are available in the context of dual careers, there is comparatively little focus on soccer, the most prominent sport in many European countries. This is especially true for professional women's soccer. An analysis of vocational and academic education that is taken up during the top-level sporting career is particularly relevant for women's soccer that, on the one hand, receives more media attention than most Olympic sports but, on the other hand, offers far lower earning opportunities than male soccer. Accordingly, it is important to empirically analyze the value that female players attach to educational investments and which behaviors go hand in hand with this.

## 3 Theoretical framework

### 3.1 Educational decisions in the context of human capital theory

The fundamental decision as to whether female soccer players pursue parallel vocational or academic education during their top-level sporting career can be modelled in the context of human capital theory (47). This theory assumes that investments in human capital (e.g., through academic or vocational education) lead to an increase in productivity, which in turn leads to a higher attainable income (48). Education, qualification and learning activities in this sense represent individual investment in the performance potential (human capital) that can be utilized on the labor market (47). Individuals invest in education (vocational or academic) in order to earn higher incomes in the future after discounting the education (see critical discussion from 49, among others). Such investment decisions in human capital are linked to expected (opportunity) costs and expected income or career opportunities. Accordingly, those job-related education options are chosen that promise the greatest career opportunities and associated income returns for the costs incurred (time, money) (47).

Despite their increased popularity, female soccer players in Germany only earn a low income from their sport. Surveys (although not representative) show that not even 20% of German female soccer players in the Women's Bundesliga and 2nd Women's Bundesliga earn a monthly salary of more than € 1,000 (50). If one follows the core assumptions of human capital theory, then it can be assumed that the majority of female soccer players will benefit (in the long term) from investments in vocational or academic education due to the comparatively low returns on income in women's soccer (3). If female soccer players consider future income and labor market opportunities after their sports career to be important, they will push for professional qualification because the expected income appears to them to be favorable for amortizing the necessary educational expenses (time, money).

However, the social structure of elite sport in general and soccer in particular is characterized by a strong orientation on the present (20). Top athletes usually exhibit a strong biographical fixation on elite sport, which leads to discounting of the future (on the concept of hyperbolic discounting, see 51). Consequently, other career areas and corresponding activities (e.g., professional education) may be neglected (52). The willingness of female soccer players to continue with their professional education, which is an individual investment decision beyond the end of their sporting career and thus over a longer planning horizon, thus collides with the players' orientation toward the present and influences their value structure. Such a time preference can lead to an extension of time investments in sports and to a restriction or complete neglect of education-related efforts.

### 3.2 Factors influencing starting and completing academic or vocational education

Female soccer players operate in the setting of elite sport, which is why *soccer-related factors* have to be included in the analysis. Within elite sport, sporting success is the primary goal (53). On the one hand, there are likely to be players who accept neglecting education to increase or secure their chances of sporting success, as professional qualifications can still be gained or upgraded after the end of their sporting career. On the other hand, other players will take advantage of academic or vocational education options during their sporting career to secure alternative professional career prospects for their future livelihood (7). If players have the expectation that a parallel education will have a negative impact on soccer, then it can be assumed, especially for successful female soccer players, that they will forego an education or postpone completion of it (35). With increasing sporting success, the opportunity costs of taking up vocational or academic education rise. If sporting success suffers as a result of the necessary investment in vocational or academic education, this is associated with financial losses and loss of reputation, especially for successful female soccer players.

It should also be noted that female soccer players only have a limited time budget. Due to training, competition, physiotherapy and other appointments, female soccer players are often heavily involved in sport (28, 30). Professional sport as a full-time job (53) can limit opportunities for pursuing vocational or academic education.

Educational decisions of female soccer players can also be influenced by the resources acquired during their elite sport career. For example, existing findings (e.g., 54) show that athletes with high social recognition and prominence are less likely to invest in a dual career. This is explained by the fact that there is no compelling financial necessity (35) and a later professional position in the field of sport is more likely to be secured (55). Accordingly, an (overly) optimistic assessment (overestimation) of the usability of resources accumulated in the course of a sports career (symbolic capital/reputation, see 56) could have a negative impact on the assessment of the value of academic or vocational education, which is why the influence of familiarity must be included in the analysis. It is also important to analyze the extent to which the expectation that skills acquired in elite sport (“transferable skills”) (57, 58), are advantageous for starting a career has an impact on decisions and trajectories related to academic or vocational education.

It should also be examined to what extent the possible end of a soccer career influences starting and completing academic or vocational education by shifting cost-benefit relations (59). Female soccer players who are close to the anticipated end of their sporting career may make different utility trade-offs than players at the beginning of their sporting career.

In addition to soccer-related factors, *social and socio-demographic factors* must also be considered. It can be assumed that the willingness of female soccer players to pursue vocational or academic education increases with increasing support (31, 35, 60). Advice from, for example, career counselors, family members, friends or players' agents can raise players' awareness of the importance of vocational or academic education and reduce search and information costs in relation to suitable education opportunities (61).

With increasing age, the risks of a lack of education increase, as it is hardly possible to compensate for the income lost by starting a career (too) late (28). Existing studies suggest that top athletes focus on their educational careers more strongly from the age of 25 at the latest (62).

Since individual stages within school, academic or vocational education influence each other in their chronological sequence (63), the school-leaving qualification must be considered. This shows that further education in the form of a degree is easier for top athletes with a higher education entrance qualification in comparison to athletes who want to pursue vocational education (64).

It is also important to examine the extent to which nationality influences academic or vocational education. Foreign players who play in the Bundesliga or the 2nd Bundesliga in Germany are likely to be highly success-oriented and identify strongly with top-level sport (65). It can be assumed that the opportunity costs of vocational or academic education are high for these players, which may reduce their willingness to pursue it (38). In addition, language barriers and a lack of familiarity with the German education system can have a negative impact on the willingness to pursue vocational or academic education.

## 4 Method

### 4.1 Sample

From November 2021 to January 2022, an online survey was conducted among female soccer players in the German Women's Bundesliga and 2nd Women's Bundesliga. To create the questionnaire, a pre-test (*n* = 5) was carried out with active female soccer players from the third highest league in Germany (Regionalliga). There was no need to change the content of the questionnaire after the pre-test. After data cleaning, a total of *n* = 200 questionnaires (German: = 191; English: *n* = 9) were included in the analysis, which corresponds to approx. 29.6% of the addressed population. The average age of the players was 23.3 years, which is lower than the average age of the total population in both leagues of 24.3 years. This difference is because female players from the Bundesliga, who are on average older than female players from the 2nd Bundesliga, are (slightly) underrepresented in the sample (35.5%) in relation to the relevant population (42.6%) (Table 1).

**Table 1:** Description of the sample in relation to the population.

	Population		Sample	
	*N*	%	*N*	%
League				
Bundesliga	288	42.60	71	35.50
2nd Bundesliga	388	57.40	127	63.50
Not assignable	0	0	2	1.00
Total	676	100.00	200	100.00
Nationality				
German	539	79.70	181	90.50
Not German	137	20.30	19	9.50
Total	676	100.00	200	100.00
A-National Team				
Bundesliga	107	37.20	23	32.40
2nd Bundesliga	10	2.60	2	1.60

In addition, players who are German nationals are overrepresented in the sample, while female players from A-National Teams (of different nations) are (slightly) underrepresented (Table 1).

### 4.2 Measurement

To analyze the educational status, it was asked whether and, if so, in what form the female soccer players had already completed professional education (academic, vocational) or whether and, if so, in what form they are currently pursuing an education (school, academic, vocational). For further bivariate and multivariate analyses regarding factors associated with willingness to pursue vocational or academic education, the willingness to pursue an education was operationalized dichotomously (completed or currently in vocational or academic education vs. no completed and not currently in vocational or academic education). Likewise, the completion of vocational or academic education was operationalized dichotomously (completed vocational or academic education vs. no completed but currently in vocational or academic education). In order to test the possible influence of soccer on vocational or academic education, the players were asked the following question on a 5-point scale from 1 (substantially worsened) to 5 (substantially improved): *According to your own assessment, in what way has soccer influenced your vocational or academic education?* The question was to be answered in terms of both educational performance and education duration.

The independent variables were operationalized as follows: sporting success was mapped via league affiliation (Bundesliga vs. 2nd Bundesliga) and membership of the A-National Team (yes vs. no). The time commitment to soccer was considered by asking the number of hours that the players spend on soccer on average per week (excluding competitions) (28). The current relevance of a career end was assessed on a 5-point scale from 1 (not at all) to 5 (very intensively) using the following question: *How intensively are you currently dealing with the end of your top sporting career?* For mapping a possible influence of self-perceived familiarity (following 30) in connection with the expected benefit of familiarity when entering a profession, both aspects were first asked separately. Familiarity was assessed on a 5-point scale from 1 (not at all known) to 5 (very well known) using the following question: *How well known do you consider yourself to be in Germany within your sport?* The possible benefits of familiarity for professional career entry were asked on a 5-point scale from 1 (strongly disagree) to 5 (strongly agree) as follows: *To what extent do you agree that being known will help when starting a professional career?* Subsequently, by multiplying the two items, the factor “advantages of being known when starting a professional career” was formed for further analyses.

Expectations of the usefulness of transferable skills (57) for career entry were assessed on a 5-point scale from 1 (strongly disagree) to 5 (strongly agree) using the following question: *To what extent do you agree that top athletes are preferred by employers because of specific characteristics they are believed to have, such as resilience, determination and the ability to work in a team?* The perceived support with regard to vocational or academic education issues was asked on a 5-point scale from 1 (not at all) to 5 (very intensively) as follows: *To what extent are you advised by the following parties in professional education-related matters?* The players had to answer this question for the following parties: family, friends, career advisors at the Olympic training centers, club/federation, German Sports Aid Foundation, players' agents. For the further analyses, an overall factor (mean) of “advice concerning vocational or academic education” (*α* = 0.63) was formed. The (first) nationality was asked openly and coded dichotomously (German vs. other nationality). The highest school-leaving qualification was asked in a differentiated manner. After excluding the schoolgirls, the variable was coded dichotomously (higher education entrance qualification vs. other school-leaving qualification).

### 4.3 Data analyses

First, descriptive analyses were implemented on the status of vocational or academic education and the influence of soccer on vocational or academic education. Subsequently, bivariate analyses (Mann-Whitney U test; Pearson chi-square test) were carried out to test the influence of various factors on the willingness to pursue vocational or academic education. In a next step, a logistic regression model was estimated to take into account relevant factors that influence the attainment of a qualification. It should be noted that the respective effects within the nested model should not be interpreted and compared using the coefficients or odds ratios (ORs) (66, 67). Instead, the average marginal effects are calculated. The average marginal effect (AME) expresses the average influence of the independent variable on the probability of occurrence *P* (*y* = 1|*x*) in a single index' (68). Multicollinearity was tested for the regression model. The variance inflation factor (VIF) did not have values higher than 4.0, which means that there is no multicollinearity between the individual explanatory variables. The number of cases per predictor was regarded as good (69). There were only a few numbers of outliers in the dataset (*n* < 5%) (all standardized residuals were −2,59 ≤ SResid ≤ 2,14) (70);. All continuous predictors were found to follow a linear relationship to the logit of the dependent variable (using the Box-Tidwell procedure) (71);. Nagelkerke's pseudo R² and the Hosmer-Lemeshow adaptation test were reported for all models.

## 5 Results

### 5.1 Descriptive findings on education status and the influence of soccer

Of the 200 female soccer players, 20.5% (*n* = 41) were still schoolgirls at the time of the survey. The remaining 79.5% (*n* = 159) had completed their school education. For the analyses on pursuing and completing vocational or academic education, the schoolgirls were excluded from the analysis, as pursuing vocational or academic education is not yet relevant for them.

The educational status of the female soccer players who already have a school-leaving certificate (*n* = 159) can be seen in Table 2. 29.0% of the players have (at least) one academic degree and 20.1% have (at least) completed one vocational education. 36.5% of the players are currently studying without a preliminary academic degree or completed vocational education. If we also include the players who have already completed (at least) one academic or vocational education and are pursuing (further) studies (18.3%), then 54.8% of the female soccer players were studying at the time of the survey. 5.0% of female soccer players are in vocational education without a preliminary academic degree or completed vocational education. 9.4% of the players are neither studying nor in vocational education and have no academic degree or completed vocational education.

**Table 2:** Professional education status.

	%	*N*
Completed academic education	16.40	26
Completed academic education and currently in further academic education	12.60	20
Currently in academic education	36.50	58
Completed vocational education and currently in further academic education	5.70	9
Completed vocational education	14.40	23
Currently in vocational education	5.00	8
No academic degree or completed vocational education and currently not in vocational or academic education	9.40	15
Total	100.00	159

The transition rate—in terms of the ratio of female soccer players who are eligible to study to the players who take up studies—is 83.7%. In addition, a further 5.6% of female soccer players with a general higher education entrance qualification plan to start studying within the next 1 to 3 years.

With regard to the influence of the sporting career on vocational or academic education, 18.1% of the female players, report that soccer has (substantially) worsened their performance in vocational or academic education. 61.7% of the respondents report no influence while 20.2% report a (substantial) improvement. A (substantial) worsening of the duration of vocational or academic education is stated by 30.5% of the players, while 59.1% of the respondents report no influence. 10.3% of the female soccer players, state that the duration of their vocational or academic education has (substantially) improved as a result of soccer (see Table 3).

**Table 3:** Influence of soccer on vocational or academic education.

	%	%	%	%	%	M	SD	*N*
Influence of soccer on vocational or academic education (5-point Likert-scale 1 = substantially worsened, 5 = substantially improved)	1				5			
	0.60	17.50	61.70	13.70	6.50	3.08	.77	154
Influence of soccer on duration of vocational or academic education (5-point Likert-scale 1 = substantially worsened, 5 = substantially improved)	1				5			
	9.70	20.80	59.10	7.10	3.20	2.73	.86	154

### 5.2 Bivariate findings on willingness for starting vocational or academic education

It is important to emphasize that, as shown in Table 2, 90.6% of the female soccer players are currently pursuing or have already completed vocational or academic education. Since only 9.4% of the players have not (yet) shown any willingness to pursue such education, the influence of possible factors on this willingness is estimated bivariate and not by means of a regression analysis.

As the results in Table 4 show, significant differences between the two groups can only be found for age and nationality. Female soccer players who are in vocational or academic education or have completed it are on average 24.92 years old, while players who are not in vocational or academic education are on average 21.27 years old. The influence has a medium effect size. Regarding nationality, it is evident that players with non-German nationality belong disproportionately to the group of players without a willingness to pursue vocational or academic education (26.67% vs. 9.40% share in the total sample). There is a (rather) weak correlation.

**Table 4:** Bivariate findings on willingness for vocational or academic education.

	Completed and/or currently in vocational or academic education (*n* = 144)			No completed and not currently in vocational or academic education (*n* = 15)			Total (*n* = 159)			Statistics
	%	M	SD	%	M	SD	%	M	SD	
League affiliation										
Bundesliga	41.95			42.85			42.00			*χ*²(1) = .004; *p* = .948
2nd Bundesliga	58.05			57.15			58.00			
A-National Team										
Yes	14.58			33.33			16.40			χ²(1) = 3.492; *p* = .062
No	85.42			66.67			83.60			
Average time spent on playing soccer per week (without competition)		13.31	4.96		13.53	5.45		13.33	4.99	*U* = 1,059.500; *p* = .904
Thought of ending one's soccer career		2.72	1.26		2.20	1.32		2.67	1.27	*U* = 826.000; *p* = .124
Expected advantages of being known when starting a professional career		8.04	6.51		9.46	7.54		8.17	6.59	*U* = 811.000; *p* = .621
Expected advantages when starting a professional career through characteristics of top athletes		3.47	.88		3.43	.94		3.46	.89	*U* = 907.000; *p* = .722
Advice concerning vocational or academic education		2.09	.57		2.21	.30		2.10	.55	*U* = 704.000; *p* = .081
Age		24.92	3.86		21.27	3.08		24.57	3.94	*U* = 429.500; *p* = <.001; *r* = 0.305
Nationality										
German	92.36			73.33			90.60			χ²(1) = 5.757; *p* = .016; V = 0.190
Other nationality	7.64			26.67			9.40			
School-leaving qualification										
Higher education entrance qualification	86.11			80.00			85.50			χ²(1) = .410; *p* = .522
Other school-leaving qualification	13.89			20.00			14.50			

### 5.3 Multivariate findings on the completion of vocational or academic education

Logistic regression analysis (Table 5) was used to estimate which factors have an influence on whether a vocational or academic education could already be completed. Both the Hosmer-Lemeshow test: *χ* (8) = 5.940, *p* > .05 and Nagelkerke's pseudo *R*^2^ = .691 indicated a good model fit (72, 73). Thus, the model correctly predicted 84.6% of all cases. However, the confidence interval of some factors has a very high range, which makes it difficult to estimate the “true” value of the respective effect size.

**Table 5:** Influencing factors on completed academic or vocational education (multiple logistic regression; odds ratios (OR), average marginal effects (AME) and the confidence interval in brackets are reported).

	Model	
	OR	AME
Soccer-related factors		
League affiliation (ref. Bundesliga)		
2nd Bundesliga	1.111 [0.248; 4.987]	0.011 [−0.144; 0.166]
A-National Team		
No	118.573 [4.687; 3,000.018]**	0.351 [0.236; 0.466]**
Average time spent on playing soccer per week	0.837 [0.725; 0.967]*	−0.018 [−0.032; −0.005]*
Thought of ending one's soccer career	0.910 [0.555; 1.492]	−0.010 [−0.061; 0.041]
Expected advantages of being known when starting a professional career	1.097 [0.955; 1.261]	0.010 [−0.005; 0.024]
Expected advantages when starting a professional career through characteristics of top athletes	1.880 [0.954; 3.705]	0.065 [−0.002; 0.132]
Social & Sociodemographic factors		
Advice concerning vocational or academic education	2.738 [0.773; 9.697]	0.104 [−0.021; 0.229]
Age	2.820 [1.938; 4.103]***	0.107 [0.094; 0.121]***
Nationality (ref. German)		
Other nationality	0.392 [.0.013; 11.669]	−0.094 [−0.423; 0.234]
School-leaving qualification (ref. Higher education entrance qualification)		
Other school-leaving qualification	478.846 [25.404; 9,025.955]***	0.461 [0.385; 0.537]***
Nagelkerkes Pseudo *R*^2^	.691	
Hosmer-Lemeshow test	.654	
*N*	136	

* Significance level: *p* < .05.

** *p* < .0.

*** *p* < .001.

If we look at the influence of soccer-related factors, we see a strong correlation depending on membership of the A-National Team. For players who are not members of the A-National Team of their respective country, the probability of having already completed their academic or vocational education increases by 35.1% (AME = 0.351). It is also clear that players who are more involved in soccer have a lower probability of having already completed their professional education. On average, the probability decreases by 1.8% (AME = −0.018) per additional hour spent on soccer per week.

There is no significant influence from the factors of “league affiliation”, “thought of ending one's soccer career”, “expected advantages when starting a professional career after soccer through being known” and “expected advantages when starting a professional career after soccer through characteristics of top athletes”.

In terms of social and socio-demographic factors, there is a strong influence of age. If the age of the players increases by one year, the probability of having completed their academic or vocational education increases by an average of 10.7% (AME = 0.107). In addition, female soccer players who do not have a higher education entrance qualification are 46.1% (AME = 0.461) more likely to have successfully completed their professional education than female soccer players with a higher education entrance qualification.

The other factors included in the analysis—counseling and nationality—do not have a significant influence.

## 6 Discussion

### 6.1 Discussion of the results and contribution to the literature

The present study analyses the educational careers of elite female soccer players and thus follows on from the question of the extent to which soccer poses a risk for professional career after the sport career (22). This is because, in addition to financial resources and sporting success, educational qualifications and educational or vocational skills have an important influence on the quality of the transition from an active sports career into a post-sports career (74, 75). Thus, the present study responds to calls for a more athlete-centered approach in sports science research (76–78). Furthermore, the article focuses on elite female athletes. It is true that women have made substantial progress in participating in elite sport. However, sport is not free from gender discrimination (79). Professional women's sport in general, and professional women's soccer in particular, is often (still) seen as second-class and less important compared to men's sport (80). Likewise, many areas of sport science research show a gender imbalance in terms of inclusion in studies (81). By focusing on top-level women's soccer, this paper increases the visibility of women as a subject of analysis in sports science research in general and in relation to dual careers in particular. In addition, the level of knowledge regarding of academic and vocational education during the soccer career will be expanded. This addresses the sport of soccer which, despite its prominence and popularity, has often been neglected in previous studies on dual careers.

The following key findings can be deduced from the available results of the study: the players in the Women's Bundesliga and 2nd Women's Bundesliga show a very high willingness to pursue an education. Only 9.4% of female soccer players with a school-leaving certificate were not pursuing vocational or academic qualification at the time of the survey and had not yet completed a vocational or academic education. This means that most players opted for a dual career path (42) by pursuing or having pursued vocational or academic education parallel to their top-level sporting career. The high value of vocational or academic education has already been demonstrated among professional female soccer players in England (7). The willingness of female soccer players to pursue vocational or academic education is significantly higher than that of their male counterparts. As Mazurkiewicz (23) was able to show, almost half (47.6%) of soccer players in the 3rd and 4th leagues in Germany do not have a professional qualification and are not pursuing vocational or academic education. It is also striking that 71.2% of female players are studying or have already completed their studies, while the proportion among their male colleagues is only 28.9% (25). It should be noted that male soccer players in the 3rd and 4th leagues in Germany have a relatively lower level of performance compared to the female soccer players in the Women's Bundesliga and 2nd Women's Bundesliga but generate a (significantly) higher income from their sport. The average monthly income of male soccer players in the 3rd league is €10,000 (82), whereas not even 20% of female soccer players in the Women's Bundesliga and 2nd Women's Bundesliga earn a monthly salary of more than €1,000 (50). Higher financial income may mean that taking up vocational or academic education is considered less necessary for financial reasons (35) and/or the opportunity costs of vocational or academic education increase, which can also have a negative impact on the willingness to pursue education (47). The notion that the willingness of female soccer players to pursue vocational or academic education would decrease if the financial reward increased seems possible from the perspective of human capital theory (47). However, this remains speculative because, based on our data, the analysis cannot provide a valid answer.

The results also show that a higher proportion (29.0%) of female soccer players already have an academic degree compared to top athletes in Germany in general (23.7%, 28). It is also clear that the transition rate—in the sense of the ratio of those eligible to study to the actual students (so far)—of 83.7% is at a higher level compared to the total female population in Germany (77.0%, 83). The findings thus suggest that most female soccer players opt for vocational or academic education that corresponds to their school-leaving level and do not choose inadequate or low-return career paths (84).

The majority (61.70%) of female soccer players also report that soccer has or had no impact on performance in vocational or academic education. 20.2% even report a positive impact of soccer on educational performance. For some female soccer players, positive synergistic effects can even be observed. Thus, top-level sport does not necessarily have a negative impact on vocational or academic performance, as has been shown in other studies (27–29). A negative influence of soccer on performance in vocational or academic education was reported by (only) 18.1% of female soccer players. Fundamental problems with the temporal and organizational coordination between soccer and vocational or academic education, as identified among Danish female soccer players (24), cannot be identified based on our present findings. A possible reason for the overall good compatibility between soccer and vocational or academic education may lie in the comparatively moderate amount of time that the female soccer players have to invest in training. The average of 13.2 h a week is below the value of 18.4 h that German squad athletes in general have to invest on average in weekly training (28). In addition, the majority of female soccer players complete an academic education and, in comparison with vocational training, a sporting career can be better combined with studying (37, 64). Studying can be spread out over time, which reduces the average weekly load, although athletes need more time to complete their studies (28). Furthermore, other empirical findings suggest that elite sport and vocational or academic education can have a positive influence on each other (27, 85). For example, because success in one area reduces the pressure to perform in the other area or because skills learned in soccer, such as determination and willingness to perform, can help you complete vocational or academic education (27, 86).

With regard to soccer-related factors that influence the willingness to pursue or complete vocational or academic education, no difference can be identified between players in the Bundesliga and 2nd Bundesliga. In contrast, female A-National Team players show a lower willingness to pursue vocational or academic education compared to female soccer players who do not belong to the A-National Team (although not significantly) and a lower proportion have completed their education to date. This is not surprising, as the opportunity costs of education increase for female national team players if this has a negative effect on top-level sporting development (47), whereby the mere assumption of a negative influence is sufficient (35). National players are therefore more willing not to pursue vocational or academic education for the time being and/or to stretch it out. The comparatively moderate workload (13.3 h) that female soccer players have to spend on sport per week on average (excluding competition) compared to German squad athletes in general (18.4 h, 28) has no influence on their willingness to pursue vocational or academic education. However, a greater time commitment to soccer results in a higher proportion of education not yet having been completed, although the delay effect proves to be comparatively small. The thought of ending a career has no influence on vocational or academic education. For female soccer players, vocational or academic education is an important precautionary strategy or back-up plan (87) for the time after their sporting career, which does not only become important when the immediate end of their career is imminent. This finding is consistent with existing findings in women's soccer (7). However, this contradicts findings from other studies on elite sport, according to which athletes only start to think more concretely about post-sport career plans towards the end of their competitive sports career (88, 89). A possible reason for female soccer players' early focus on a dual career could, in addition to comparatively low earning potential (3, 50), also be the value of vocational or academic education. Given the very high proportion of students among female soccer players, it can be assumed that they come from middle or higher social classes. In Germany, educational careers are significantly influenced by social background (90). Educational research has empirically proven that people whose parents have a higher social status attribute a high level of benefit to professional qualifications (transgenerational status confirmation) (91, 92), which can lead to female soccer players starting vocational or academic education early. In addition, the results show that whether female soccer players consider themselves to be comparatively well-known in their sport and also assume advantages of being well-known when starting a professional career after soccer has no significant influence on the willingness to pursue or complete vocational or academic education. This also applies to the aspect of whether female players assume that top athletes have advantages when starting a professional career after soccer due to specific positive characteristics attributed to them by the employer. As female soccer players do not expect their soccer career to have a direct positive influence on their professional career, this is also likely to contribute to the fact that a high proportion of female soccer players take up—as an important precautionary strategy—vocational or academic education at an early stage. Accordingly, it should also be noted that soccer coaching qualifications were not explicitly mentioned by the soccer players in relation to vocational education. It is unclear whether the coaching qualification is not regarded by the female soccer players as a “fully-fledged” professional qualification, or whether a coaching license has not (yet) been obtained. In contrast to tennis, where a high proportion of both men and women acquire coaching licenses in order to work as a coach or head of a tennis school after their sporting career (93), a comparable professional career does not appear to be very attractive for female soccer players (94). Unlike in tennis, there are hardly any jobs for coaches at grassroots level and in recreational soccer. In addition, in Germany, coaching positions in top-level sport in general and in soccer in particular are primarily filled by men, and the proportion of female coaches in top-level sport in Germany is only around 13% (95). The lack of career prospects in soccer due to existing barriers (94) is likely to further increase the need for female soccer players to focus on a dual career at an early stage.

Finally, if we reflect on the influence of social and socio-demographic factors, a strong effect of age emerges regarding the willingness both to pursue and complete vocational or academic education. Age is a “natural” predictor of when top athletes take up and complete vocational or academic education, especially in the age range between 16 and 25 years (96). Female soccer players who have a nationality other than German show a lower propensity to pursue vocational or academic education. These players, who for example move from countries such as Austria, Switzerland or Serbia to the German Bundesliga or 2nd Bundesliga, find fewer professional leagues in their countries. It can therefore be assumed that primarily sporting (and financial) reasons are decisive for the switch, which can lead to other areas of life (in this case vocational or academic education) being neglected because it is assumed that this is the only way to achieve maximum sporting success (97, 98). Nationality has no influence on the completion of education. However, the number of players included who do not have German citizenship is small, so that no reliable statements can be made. The type of school-leaving qualification has no influence on the willingness to pursue vocational or academic education. In contrast, a higher proportion of female soccer players without a higher education entrance qualification have already completed their vocational or academic education compared to players with a higher education entrance qualification. Since female players without a higher education entrance qualification are more likely to pursue vocational or academic education, which is associated with shorter periods compared to university studies, this finding is to be expected. The intensity of advising with regard to dual career issues has no influence on the willingness to pursue or completed vocational or academic education. This is surprising insofar as, for example, the support of family and peers are seen as important resources (also) in relation to dual career issues (60, 99). One possible reason could be that due to the overall very high willingness of female players to pursue vocational or academic education, the factor of support services regarding questions of dual career planning loses relevance for female soccer players compared to other top athletes.

### 6.2 Practical implications

For organized sport, the following conclusions can be drawn from the findings: (i) Female soccer players are far ahead of their male colleagues in terms of willingness to pursue vocational or academic education and already completed education. Even in comparison with top athletes in other Olympic sports with similar, sometimes even lower, earning potential, the willingness to pursue education can be considered very high. This should be taken up (more strongly) by representatives of organized women's soccer and integrated into media reporting. Top sporting success combined with successful educational careers appears to be a suitable topic to differentiate from high-income men's soccer and to counteract inequality in reporting (100), although this will only be possible to a limited extent due to the media dominance of men's soccer. (ii) Against the background of the high willingness to pursue vocational or academic education, it seems advisable for clubs and federations to support this accordingly and to counteract coordination difficulties between top-level sport and professional education. This is in the own interest of soccer clubs and federations to counteract the danger of talented female soccer players leaving the game early due to coordination problems (24, 101). The focus here should be on the early career phase in particular, when there is the greatest risk of vocational or academic education being neglected. Especially for female players on the threshold of elite soccer, support for dual careers is necessary to buffer the risks of a soccer career that can end involuntarily and sooner than planned, e.g., due to injuries (102). However, previous studies indicate rather limited support from clubs (7). (iii) In the context of this study, advising has no influence on players' decisions to pursue vocational or academic education or its course. It is noticeable that the players generally hardly ever seek advice on education-related matters. Greater involvement of experts (in Germany, for example, the career advisors at the Olympic training centers) seems advisable, as they usually have specific knowledge and networks in education-related matters.

### 6.3 Limitations and future research

Future studies must take the limitations of the present study into account. In the present study, (i) an adequate sample could be generated both quantitatively (sample comprises almost 30% of the population) and qualitatively (good representativeness). However, female players with non-German nationality are underrepresented in the sample. One possible reason for this is that the questionnaire had to be answered “only” in German and English. In follow-up studies, the survey instrument should be made available in other languages. (ii) The female soccer players fundamentally show a very high willingness to pursue vocational or academic education. This means that it is only possible to analyze influencing factors to a limited extent due to the low variance in response behavior. To increase the heterogeneity of the sample, the analysis should be extended to other international leagues in women's soccer. (iii) The findings of the present study indicate that female soccer players succeed well in combining top-level sport and vocational or academic education. However, it was not investigated which concrete efforts players have to make and potentially also which hardships they have to accept in order to achieve this. Further studies should therefore focus on the effort and coping strategies required to complete a dual career. (iv) The present study provides indications that the level of performance or sporting success, in the sense of being a member of the A-National Team, has an influence on the willingness to pursue vocational or academic education and the course of this education. For further studies, it is advisable to operationalize sporting success in a more differentiated way. (v) Research shows that a very strong sporting identity, especially if it marginalizes other identities and values (for example, regarding education), is often associated with increased risks for the person (103, 104). Future studies on dual careers in women's soccer should therefore directly analyze the influence of Athletic Identity (105, 106) on education-related decisions by operationalizing and surveying Athletic Identity in the survey instrument Brewer. (vi) Vocational or academic education are intertemporal allocation decisions. Insofar as players break down the career period over which they make educational decisions into different intervals, the time component is considered differently in the evaluation of the educational benefit (subadditive discounting, 107). Therefore, it is important to empirically analyze how female soccer players deal with these diverging time horizons, both in terms of the present (sports career) and the future (further vocational or academic education decisions). Of particular interest is whether, and in what way, the post-sport future is already considered in the players' current actions and by which factors this is in turn influenced.

## Data Availability

The raw data supporting the conclusions of this article will be made available by the authors, without undue reservation.
